# Variation in use of antipsychotic medications in nursing homes in the United States: A systematic review

**DOI:** 10.1186/s12877-017-0428-1

**Published:** 2017-01-26

**Authors:** Hannah Cioltan, Samah Alshehri, Carol Howe, Jeannie Lee, Mindy Fain, Howard Eng, Kenneth Schachter, Jane Mohler

**Affiliations:** 1 College of Public Health, University of Arizona, Tucson, Arizona USA; 2 Arizona Center on Aging, College of Medicine, University of Arizona, Tucson, Arizona USA; 3 College of Pharmacy, University of Arizona, Tucson, Arizona USA; 4 University of Arizona Health Sciences Library, Tucson, USA; 5 Division of Geriatrics, General Internal Medicine and Palliative Medicine, College of Medicine, University of Arizona, Tucson, Arizona USA

**Keywords:** Antipsychotics, Nursing homes, Dementia, Behavioral symptoms

## Abstract

**Background:**

The use of antipsychotic medications (APMs) in nursing home residents in the U.S. is an increasingly prominent issue and has been associated with increased risk of hospitalization, cardiovascular events, hip fractures, and mortality, among other adverse health events. The Food and Drug Administration has placed a black box warning on these drugs, specifying that they are not meant for residents with dementia, and has asked providers to review their treatment plans. The purpose of this systematic PRISMA (Preferred Reporting Items for Systematic Reviews and Meta-analyses)-based review was to summarize original research studies on facility level characteristics contributing to the use of antipsychotics in nursing homes across the United States, in order to investigate the variation of use.

**Methods:**

We searched Ovid Medline, Embase, Cochrane Library, Web of Science, CINAHL, PsycInfo, and Sociological Abstracts. Articles were selected according to the following criteria: (1) Population of interest: older adults (≥60 years of age) residing in nursing homes (not home-based or inpatient hospital settings) in the U.S. (2) Receiving APMs, typical and/or atypical. Specifically excluded were studies of psychotropic medications such as antidepressants, benzodiazepines, anxiolytics, hypnotics, mood stabilizers, and stimulants. All study designs were considered, though reviews, editorials, letters to the editor and opinion pieces were excluded. An expert consultant panel was consulted to categorize facility characteristics into domains and determine possible etiologies of APM use based upon each characteristic.

**Results:**

Nineteen observational studies, both quantitative and qualitative, published from 2000 to 2015, met full inclusion criteria and were included in this review. APM use varied based on multiple facility characteristics across several domains: 1) physical, 2) staffing, 3) occupancy, 4) market, and 5) quality.

**Conclusions:**

Variation in use of APMs in U.S. nursing homes based upon facility characteristics exemplifies the need for a more systematic protocol guiding the use of these medications, along with heightened regulatory policies and enforcement.

**Electronic supplementary material:**

The online version of this article (doi:10.1186/s12877-017-0428-1) contains supplementary material, which is available to authorized users.

## Background

The 1987 Omnibus Budget Reconciliation Act’s Nursing Home Reform Law, was enacted to improve patient-centered nursing home care quality, and included standards regarding freedom from unnecessary drugging; freedom from chemical restraints; and rights to be informed about, participate in, and refuse treatment [1].In 2005, the Food and Drug Administration (FDA) placed a black box warning on antipsychotic medications (APMs) use in elderly patients because of the dangerous health outcomes associated with use including mortality [2]. In a 2011 report from the Office of Inspector General, it was determined 83% of Medicare claims for APMs in nursing home residents were associated with off-label conditions [3]. Following this, in March 2012, the Centers for Medicare and Medicaid Services (CMS) launched an education and training, and oversight and provider accountability initiative targeted to provide appropriate, resident-centered dementia care to decrease the use of APMs in nursing homes nationwide by 15%. CMS added two quality measures on the Nursing Home Compare website related to APMs including the percent of long-stay residents who received an APM [4]. Despite these gains, APM use in elderly nursing home patients has continued, with 1 in 5 nursing home residents treated with APMs [5]. This problem will only grow in magnitude if not addressed. By 2050 the population of older adults aged 65 and older is projected to reach 83.7 million, almost doubling the estimated 2012 elder population of 43.1 million [6]. In 2012 15,700 nursing homes served 1.4 million residents; the number of elders residing in nursing homes will only increase with our increasing older population [7].

APMs are approved by the FDA for the treatment of schizophrenia and/or bipolar disorder but are increasingly used to treat the behavioral symptoms of dementia, an off-label use [3]. In the years between 1995 and 2008 such off-label use more than doubled [8]. The effectiveness of APMs for off-label use to treat behavioral symptoms of dementia is not supported by strong evidence and the use of APMs in patients with dementia increases the risk of adverse health events with little evidence of effectiveness [9, 10].

The use of APMs is not only a concern in the U.S. but also internationally. In the United Kingdom, a study including 12 nursing homes found 48% of patients with dementia were prescribed APMs [11], while a provider of pharmaceutical market information showed that 20.3% of patients with dementia had a prescription for APMs [12]. A report for the Minister of State for Care Services in the United Kingdom called for a national campaign to reduce APM use, citing the limited positive and significant harm causing effects of the drugs [13]. These drugs are also commonly used in the treatment of dementia in nursing homes across western Europe with rates ranging from 12 to 59%, although the clinical guidelines and warnings from European and national drug agencies suggest lowering use in this population [14]. Initiatives to reduce the use of APMs in the treatment of patients with dementia are increasingly prevalent in the U.S. and abroad—spurred by the continued high rates of use of these agents.

Adverse health outcomes associated with APM use in the elderly population include increased risk of falls, hospitalizations, cerebrovascular events, sudden cardiac death, and mortality [15]. APM use in patients with dementia is associated with a significant increased risk of stroke (OR, 3.12), cardiovascular symptoms, edema, and vasodilatation [15]. Also, the rate of sudden cardiac death among atypical antipsychotic users compared to nonusers is higher with an adjusted incidence-rate ratio of 2.26 [16]. It is vital that we reduce unnecessary use in this population because of the association between APM use and all-cause mortality. A meta-analysis of 15 randomized control trials, with 3,353 patients randomized to APMs and 1,757 randomized to placebo, showed that death occurred more frequently during the study period in patients randomized to APMs (OR, 1.54) with a number needed to harm of 100 [17].

Although previous legislation has aimed to reduce off-label use of APMs in elderly nursing home residents, prevalence remains high. It is crucial that facility level factors associated with APM use in this population be identified and understood in order to be able to recommend interventions to decrease dangerous use in an efficient an effective manner. The purpose of this systematic review was to summarize original research studies on the facility level characteristics contributing to the use of APMs in nursing homes across the U.S.

## Methods

### Study selection

A literature review was planned and performed using methods specified in the Preferred Reporting Items for Systematic Reviews and Meta-Analyses (PRISMA) statement for reporting systematic reviews and meta-analyses however a meta-analysis was not performed [18]. Both controlled vocabulary terms (e.g., MeSH) and key words were utilized to search the following databases Ovid/MEDLINE (1946–2015); Elsevier/Embase (1947–2015); Wiley/Cochrane Library (1898–2015); Thomson-Reuters/Web of Science (1898–2015); EBSCO/PsycINFO (1880’s-2015); EBSCO/CINAHL (1937–2015); and ProQuest/Sociological Abstracts (1952–2015). Literature searches were completed on July 10, 2015. The complete Ovid/MEDLINE search strategy, analogous to the other database searches, is available in Additional file 1. Reference lists of, and citations to, the articles eventually selected from the database searches were also screened. Only English language publications were selected and, in the case of both CINAHL and PsycInfo, article types were limited to Academic Journals and Dissertations.

Included studies focused on population of interest: 1) older adults (≥60 years of age) residing in nursing homes (not home-based or inpatient hospital settings) in the U.S.; and 2) those who received APMs, typical and/or atypical. Specifically excluded were studies of psychotropic medications such as antidepressants, benzodiazepines, anxiolytics, hypnotics, mood stabilizers, and stimulants alone or in combination with APMs where the antipsychotic component could not be isolated. All study designs were considered although reviews, editorials, letters to the editor and opinion pieces were excluded. No publication or language limits were applied, though ultimately only articles in English were selected. Nursing homes were defined as facilities recognized by the Medicare and Medicaid systems offering skilled nursing care including rehabilitation and various medical and nursing procedures.

Titles and abstracts of retrieved references were screened for relevance by two independent reviewers (HC and SA), one trained in public health and gerontology, and the other in gero-pharmacy. In case of disagreements, a third reviewer (CH) cast the deciding vote. In the same fashion, the full texts of the potential studies were further analyzed by two independent reviewers (HC and SA) to see if they fully met inclusion criteria. Disagreements at this stage were resolved by consensus and in consultation with a third reviewer (CH).

### Data extraction

The reviewers (HC and SA) extracted the following data from the included articles: type and number of subjects, study design, the year of publication, journal of publication, U.S. regions included in the assessment, and the facility characteristics associated with the use of APMs. The summary outcome measure considered for this review was any association between facility characteristic and APM use.

### Quality assessment

The selection process ensured that all articles included in the analysis were from peer-reviewed journals. All observational cross-sectional or cohort studies were assessed for alignment with the Strengthening the Reporting of Observational Studies in Epidemiology (STROBE) statement to enhance rigor [19].

### Data analysis

The facility characteristics shown to have an effect on the use of APMs were categorized into domains based upon categorization found in the included studies and through iterative content analysis with expert feedback from a nursing home expert consensus group of 20 experts comprised of nursing home administrators, pharmacists, an epidemiologist, a neurologist, two geriatricians, a nursing home medical director, a nursing home nurse educator, and Healthy Brain Research Network Collaborating Centers in five states (Table 1). The facility characteristics considered were extracted from the included studies. A modified Delphi method was used to ensure structure in categorizing the characteristics into domains as well as determining the possible etiologies of APM use based on each facility characteristic contributing to use. The rationale for increased APM use was also extracted from the studies when available.

**Table 1:** Expert panel details

Expert	Profession
Jeannie Lee	Geriatric pharmacist
Howard Eng	Pharmacist/health services researcher
Jane Mohler	Gerontologist/epidemiologist
Scott Bolhack	Geriatrician/nursing home medical director
Mindy Fain	Geriatrician
Debbie Dyjak	RN educator in nursing home
Lee Olitsky	Nursing home administrator
Beverly Heasley	Nursing home administrator
University of Arizona, University of Washington, University of Pennsylvania, University of South Carolina, Oregon Health and Science University, and University of Illinois at Chicago	Healthy Brain Research Network Collaborating Centers

## Results

### Study selection

**Fig. 1:**
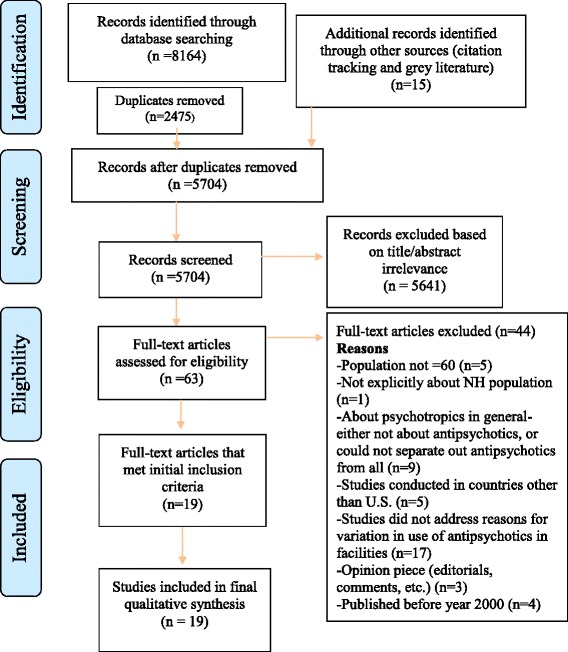
Flowchart of the process of literature search and extraction of studies meeting the inclusion criteria

We found 8,164 studies through database searches. Citation analysis of the most relevant articles revealed an additional 15 articles. Of the 5,704 articles that remained after duplicates were removed, 5,641 were excluded because of irrelevance to the topic (Fig. 1). Strict inclusion criteria, as outlined above, were applied to the full text of 63 articles. Of these, 19 met the full set of criteria (Table 2) [20–38]. All articles were published between 2000 and 2015, during which time there were several legislative, regulatory and policy changes related to use of antipsychotics as discussed in the Background Section. Please see Additional file 2 for completed PRISMA checklist.

**Table 2:** Study characteristics

Author	Study Title	Journal	Study Design	Total Pt. #	Total NH #	Sample Rep.
Bonner AF, Field TS, Lemay CA, et al., 2015	Rationales that providers and family members cited for the use of antipsychotic medications in nursing home residents with dementia	Journal of the American Geriatrics Society	Qualitative, descriptive	204	26	5 CMS regions: (III, IV, VIII, IX)
Bowblis JR, Crystal S, Intrator O, et al., 2012	Response to regulatory stringency: the case of antipsychotic medication use in nursing homes	Health Economics	Retrospective cohort	NA	14,743	48 states
Briesacher BA, Limcangco MR, Simoni-Wastila L, et al., 2005	The quality of antipsychotic drug prescribing in nursing homes	Archives of Internal Medicine	Retrospective cohort	1,096	NA	national
Briesacher BA, Tjia J, Field T, et al., 2013	Antipsychotic use among nursing home residents	JAMA	Retrospective cohort	1,402,039 & 561,681*	5,038	48 states
Castle NG, Hanlon JT, Handler SM, 2009	Results of a longitudinal analysis of national data to examine relationships between organizational and market characteristics and changes in antipsychotic prescribing in US nursing homes from 1996 through 2006	American Journal of Geriatric Pharmacotherapy	Retrospective cohort	NA	15,155 & 17,213**	national
Chen Y, Briesacher BA, Field TS, et al., 2010	Unexplained variation across US nursing homes in antipsychotic prescribing rates	Archives of Internal Medicine	Retrospective cross sectional	16,586	1,257	national
Hughes CM, Lapane KL, Mor V, 2000	Influence of facility characteristics on use of antipsychotic medications in nursing homes	Medical Care	Cross sectional	NA	14,631	national
Huybrechts KF, Rothman KJ, Brookhart MA, et al., 2012	Variation in antipsychotic treatment choice across US nursing homes	Journal of Clinical Psychopharmacology	Retrospective cohort	65,618	5,751	45 states
Kamble P, Chen H, Sherer J, Aparasu RR, 2008	Antipsychotic drug use among elderly nursing home residents in the United States	American Journal of Geriatric Pharmacotherapy	Cross sectional	11,227	1,174	national
Kamble P, Chen H, Sherer J, Aparasu R, 2009	Use of antipsychotics among elderly nursing home residents with dementia in the US: an analysis of National Survey Data	Drugs & Aging	Cross sectional	6,103	1,174	national
Kamble P, Sherer J, Chen H, Aparasu R, 2010	Off-label use of second-generation antipsychotic agents among elderly nursing home resident.	Psychiatric Services	Retrospective cross sectional	2,605	1,174	national
Konetzka RT, Brauner DJ, Shega J, et al., 2014	The effects of public reporting on physical restraints and antipsychotic use in nursing home residents with severe cognitive impairment	Journal of the American Geriatrics Society	Retrospective cohort	809, 645	4,258	6 states: CA,FL, IL,NY, OH,TX
Lester P, Kohen I, Stefanacci RG, Feuerman M, 2011	Antipsychotic drug use since the FDA black box warning: survey of nursing home policies	Journal of the American Medical Directors Association	Cross sectional (survey)	NA	250	national
Lucas JA, Chakravarty S, Bowblis JR, et al., 2014	Antipsychotic medication use in nursing homes: a proposed measure of quality	International Journal of Geriatric Psychiatry	Cross sectional	155,095	NA	7 states: CA, FL, GA, IL, NJ, OH, TX
Miller SC, Papandonatos G, Fennell M, Mor V, 2006	Facility and county effects on racial differences in nursing home quality indicators	Social Science & Medicine	Cross sectional	63,932	408	NY
Pimentel CB, Donovan JL, Field TS, et al., 2015	Use of atypical antipsychotics in nursing homes and pharmaceutical marketing	Journal of the American Geriatrics Society	Nested mixed-methods, cross-sectional study of NHs in a cluster randomized trial	93	41	CT
Stevenson DG, Decker SL, Dwyer LL, et al., 2010	Antipsychotic and benzodiazepine use among nursing home residents: findings from the 2004 National Nursing Home Survey	American Journal of Geriatric Psychiatry	Cross Sectional	12,090	1,174	national
Svarstad BL, Mount JK, Bigelow W, 2001	Variations in the treatment culture of nursing homes and responses to regulations to reduce drug use	Psychiatric Services	Longitudinal cohort	1,181	16	WI
Tjia J, Field T, Lemay C, et al., 2014	Antipsychotic use in nursing homes varies by psychiatric consultant	Medical Care	Nested cross sectional study of NHs in a cluster randomized trial	NA	60	national

*NA* not available, *NH* nursing home, *Pt* patient

Sample Rep.: sample represented in study

*overall sample and subset observed continuously for at least 90 days

**sample in 1996 and sample in 2006

### Study design and quality assessment

All 19 peer-reviewed articles were observational (either cross-sectional or cohort studies) or qualitative, from entities including: the Journal of the American Medical Association; the Journal of the American Geriatrics Society; the American Journal of Public Health; Drugs & Aging; Health Economics; Journal of Health and Social Policy; among several others. The included observational studies consisted of cross-sectional, retrospective cohort and longitudinal studies by design. The specific outcome measures included: differences of APM use in nursing homes based upon facility characteristics ([20–23, 25, 27, 31, 33, 34, 36–38]; odds ratios ([24, 28–30, 32, 33, 35]; beta values [26]; and risk ratios [25]. The articles included aligned with the STROBE statement on what should be included in a report of an observational study [19].

### Subjects and representative sample

The sample sizes ranged from 204 to 155,095 participants and 16 to 17,213 nursing homes (Table 2). All participants were nursing home residents aged 60 years or older. Many of the studies used the Online Survey, Certification and Reporting (OSCAR) database or the Minimum Data Set (MDS). MDS and OSCAR are two national systems that collect nursing home quality information required by CMS. MDS collects information about nursing home residents and OSCAR about the facility [39].

### Facility characteristics associated with APM use

Table 3 depicts the characteristics associated with use of APMs: 1) physical facility characteristics such as geographic location; 2) staffing characteristics including staff to patient ratios and type of staffing; 3) occupancy characteristics such as occupancy rate; 4) market characteristics such as the presence of competition; and 5) quality characteristics, for example, the regulatory reporting of physical restraint use.

**Table 3:** Facility characteristics associated with APM use

Factors increasing use		Probable Etiologies		References
		Expert panel inferences	Article Explanations	
Physical Facility Characteristics				
Physical Location	Located in metropolitan area	- Possible greater share of for-profit facilities - Different organizational culture in urban locations - More crowded NH may result in less medication screens -Less staff per resident	-No explanation given	Stevenson, 2010
	Not located in the West or Midwest OR Located in the central South or Northeast	- Different state laws and regulation regarding NHs -Regional variation in training/org. cultures/hiring patterns/staffing levels and mix may all affect quality of care -Difference in provider practice pattern	- Approaches may differ regionally - Facilities in the East used a psychiatrist more often than those in the West - Note: Briesacher, 2005 et al. found lower APM rates in southern U.S.	Briesacher, 2005, Briesacher, 2013, Chen, 2010, Hughes, 2000 & Stevenson, 2010
Facility Size	Smaller facility size	-Economies of scale. As a result, larger facilities may be able to have more specialization and devote greater resources to quality care/improvement	-Larger facilities may be able to provide more comprehensive services due to economies of scale and may be more able to implement change processes	Chen, 2010, Hughes, 2000 & Kamble, 2009
Business Type	For-profit status	- Maximize profit and minimize cost - APMs may substitute for staff, education or training - For profits minimize expenditure which leads to low quality of staffing - Non-teaching environment can be slower to adopt clinical guidelines	-APMs may be used to maximize profits and minimize the need for hands-on care -APMs may be used in for-profit facilities as chemical restraints	Castle, 2009, Hughes, 2000, Lester, 2011, Miller, 2006 & Lucas, 2014
Presence of Acuity Services	Alzheimer’s disease special care unit or other special care units	-The proportion of patients with Alzheimer’s disease or dementia may be larger than in other NHs -Dementia- related behavioral symptoms may occur more often	- A result of the impact of case-mix that is not completely captured in the aggregate diagnostic and behavioral variables included as controls	Hughes, 2000
Staffing Characteristics				
Staff Ratios	Lower RN Staffing	- Lower staff to patient ratios means less time spent with patients resulting in increased APM use	- Greater use of APMs has been consistently associated with lower staff to patient ratios	Hughes, 2000, Lucas, 2014, Miller, 2006, & Svarstad, 2001
	Lower nurse aid staffing	- Nurse aides spend more time with the patients, which results in less need for pharmacological treatment	- Nurse aides may have more patient time, resulting in less APM use	Hughes, 2000
	Higher LPN staffing	- Less time spent with the patients -Different level of training could play a role	- LPNs do not spend as much time with the patient	Lucas, 2014
BH Expertise	Increasing number of mental health professionals and physicians	- Physicians typically spend very little time with nursing home patients - NHs with more mental health professionals may accept more patients with BH issues	- Consultant psychiatry is often identified with higher APM use - Lucas et al. found however that the presence of mental health staff did not affect APM use	Bonner, 2015, Hughes, 2000 & Lucas, 2014
	Facilities served by the highest-ranked psychiatric consultant group	- High ranked psychiatric consultant groups make take on NHs with more BH problem patients, resulting in higher APM use	- Characteristics of psychiatric consultant groups can influence prescribing	Tija, 2014
Less SS support	Minimal involvement of social services	- Social services may caution against the use of antipsychotic medications or involve the family	- Social services influence decision making regarding antipsychotic medication use.	Bonner, 2015
Occupancy characteristics				
Resident Mix	Greater Facility share of Medicaid residents	-Lower funding results in less quality of care and increased use of APMs	- Medicaid provides less funding than private insurance resulting in fewer overall funds, possibly resulting in higher APM use -Lower Medicaid reimbursement is associated with increased APM use	Castle, 2009, Hughes, 2000, Lucas, 2014 & Stevenson, 2010
	Lower Medicare census	No explanation	No explanation given	Stevenson, 2010
	Increased racial diversity	-Less funds are associated with lower quality of care in NHs	- Less funds, less resources, aligning with the idea of two tiers of USA NH care	Bonner, 2015 & Miller, 2006
Occupancy rate	Low occupancy rate	- Maybe NHs with high APM use become less favorable for the elder population and their families	- Less funds are available and APMs may be used as a cheaper alternative for staff	Hughes, 2000
Market Characteristics				
Competition	Minimal or no presence of competition	- Competition may force NHs to improve quality of care to maintain occupancy	- The presence of competition has shown to increase the quality of care in NHs	Castle, 2009
Chain membership	Independent Ownership (not part of a chain)	- May have less resources, standardization, and accountability, which may lower quality of care	- Chain membership may result in a higher degree of corporate standardization and oversight	Castle, 2009
Quality Characteristics				
Reporting deficiencies	NH subject to reporting of physical restraints	- Facilities used chemical restraints instead of physical restraints in place of addressing root causes of the overuse	- The result of subjecting NHs to report physical restraint use was an increase of antipsychotic use as a substitution	Konetzka, 2014
Deficiency citations	Facilities with a higher number of deficiency citations	- Facilities ranked in the highest quartile for deficiencies most likely provide lower quality of care, which could result in the use of APMs as chemical restraints	-Multitasking incentive problem. The efforts to improve quality are spread to multiple areas of concern	Lucas, 2014 & Bowblis, 2012

*BH* behavioral health, *LPN* licensed practical nurse, *MD* doctor of medicine, *NH* nursing home, *RN* registered nurse

The physical characteristics associated with use of APMs were: 1) physical location, specifically whether the facility was in an urban setting or not and/or its specific geographic location in the country; 2) facility size; 3) profit status; and 4) presence of acuity services [23–26, 29, 31–33, 35]. The staffing characteristics included: 1) staffing ratios; 2) behavioral health (BH) expertise; and 3) social services support [16, 20, 26, 32, 33, 36, 37]. Occupancy characteristics involved the resident mix and occupancy rate [20, 24, 26, 32, 33, 35]. Market characteristics associated with APM use in nursing homes included competition and chain membership [24]. Finally, the two quality characteristics associated with higher use were 1) facilities subject to the reporting of physical restraints and 2) facilities with a higher number of deficiencies [21, 32, 38].

Several facility characteristics were reported more frequently than others in our included studies. The positive associations between both lower RN staffing and increasing facility share of Medicaid residents with APM use were each reported in four different studies (Table 3) [24, 26, 32, 33, 35, 36]. Also, the associations between APM use and each of the characteristics: geographical location and for-profit status were shown in five separate studies [23–26, 32, 33, 35].

## Discussion

The purpose of the current systematic review was to study the association between facility characteristics and the use of APM among older adults residing in U.S. nursing homes. Facility-based characteristics that are associated with APMs use in U.S. nursing homes may be categorized into several domains: 1) physical, 2) staffing, 3) occupancy, 4) market, and 5) quality (Table 3).

The current systematic literature review revealed many facility level characteristics that play a role in increasing the use of APMs in the nursing home population. Investigating these characteristics is important because variation based upon facility characteristics could indicate the absence of a nationally consistent and systematic approach to the use of APMs in nursing homes. In order to inform possible interventions to reduce the unnecessary use of APMs, it is important to understand the associated domains of the characteristics as well as the possible reasoning behind the increased use. The complete list of explanations for increased APM use based upon each characteristic are specified in Table 3.

### Physical characteristics

The physical characteristics associated with APM use included physical location, facility size, business type, and the presence of acuity services. Regional variation in APM use was evident with a positive association between APM use and facility location in the central south or northeast U.S. regions [23, 25, 26, 29]. However, one study by Briesacher et al., 2005 expressed a negative association between APM use and nursing homes in the southern United States [22]. It was also found that facilities in the southern U.S. tended to prescribe conventional APMs whereas facilities in the northeastern U.S. prescribed atypical APMs [27]. This positive association between APMs and facilities in the south or northeast U.S. regions could be due to different state laws and regulation or regionally differing treatment approaches of behavioral symptoms of dementia. A positive association between metropolitan location and APM use was evident and these facilities were more likely to prescribe atypical APMs [27, 35]. Increased facility size was conversely negatively associated with APM use, indicating that larger facilities may have more ability to implement change processes or provide more comprehensive services as a result of economies of scale [25, 26, 29]. Larger facilities were also more likely to prescribe atypical APMs rather than conventional [27]. Five articles included evidence that for-profit facilities were positively associated with APM use [24, 26, 31–33]. Kamble et al. found a positive association between the off-label use of APMs and non-profit facilities [30]. Finally, the presence of acuity services was positively associated with the use of APMs, possibly due to larger proportion of residents with more complex medical conditions and behavioral symptoms [26]. Huybrechts et al. found that facilities with Alzheimer special care units more often prescribed atypical APMs [27].

### Staffing characteristics

Staffing characteristics play a pivotal role in the increased use of APMs in nursing homes and include: 1) staffing ratios; 2) the presence of mental health professionals or physicians; and 3) the presence of social services (Table 3). Registered nurse (RN) staffing plays a critical role in the increased use of antipsychotics with a positive association between decreased RN staffing and APM use [26, 32, 33, 36]. Less time is available per patient when nursing staff to patient ratios are low, and APMs may serve as a cost-saving alternative to hiring additional RNs. Interestingly, licensed practical nurse (LPN) to patient ratios have the opposite effect on the use of antipsychotics. A positive relationship between LPNs and APMs exists [32]. We theorize that the difference in training between an LPN and RN could play a role in such association.

In addition to nurse staffing ratios, the presence of mental health professionals, physicians and social services all affect the use of APMs with the presence of mental health professionals positively associated with APM use [20, 26, 32]. Facilities with mental health staff were also more likely to prescribe atypical APMs [27]. This positive association could be explained by a greater percentage of residents with behavioral problems in nursing homes where mental health professionals are more available or to the practice patterns of mental health professionals and physicians. Conversely, the presence of social services was negatively associated with the use of APMs. This may be caused by increased patient-clinician face time or by the inclusion of the family in decision-making [20]. It is important to note that Lucas et al. found no association between the presence of mental health staff and APM use [32]. A complete list of staffing characteristics and etiologies of use can be found in Table 3. Overall, it appears that the more time that staff is able to spend with the patients in nursing homes, the less likely it is that APMs are used.

### Occupancy characteristics

Both the resident mix and occupancy rate of a facility contributed to variation of APM use among facilities [20, 24, 26, 32, 33, 35]. Facilities with a greater facility share of Medicaid residents were positively associated with APM use [24, 26, 32, 35]. Medicaid reimbursement rates are on average 70% of private-pay reimbursement rates and considerably influence the resources available in nursing homes [40]. Consequently, APMs may be used to minimize resource use, or be used as a result of lower staffing levels or insufficient training. Kamble et al. found, however, that Medicaid patients were negatively associated with off-label APM use [30]. Low occupancy rate was positively associated with APM use, again probably due to a decreased availability of funds to increase staffing, education and/or training [26]. It may be reasonable to assume that the quality of care goes down when facility care capacities are exceeded. Also, a lower Medicare census, increased racial diversity, and self-pay patients were positively associated with APM use according to several studies [20, 30, 33, 35]. Finally, it is important to note that one study found that payment source does not affect the use of APMs [28]. The occupancy characteristic etiologies of increased APM use were commonly related to fewer resources and limited funding.

### Market characteristics

Two market characteristics, independent ownership and minimal competition, were positively associated with use of APMs in U.S. nursing homes [24]. Competition may reduce APM use by forcing nursing homes to improve or maintain higher quality to maintain occupancy. Independent ownership may contribute to increased rates of use as a result of a lack of standardization, accountability and oversight [24]. Interestingly, Pimentel et al. found that pharmaceutical marketing was not significantly associated with the use of antipsychotics [34].

### Quality characteristics

Facilities subject to reporting physical restraint use and with higher numbers of deficiency citations were positively associated with APM use [21, 32, 38]. Requiring nursing homes to report physical restraints was positively associated with APM use, possibly due to the fact that APMs, presumed to have fewer adverse effects, were used as a substitute [38]. The number of citations received at a nursing home was also positively associated with APM use [32]. It has been previously shown that lower quality is associated with increased citations [21, 32]. It is possible that the nursing homes might have a multitasking incentive problem, wherein the efforts to improve quality are spread to many areas of concern and could lead to not adequately addressing the use of APMs [21].

### Policy and management implications

Despite multiple legislative and educational interventions, including the 1987 Omnibus Budget Reconciliation Act’s Nursing Home Reform Law, the FDA’s black box warning on antipsychotic medications, the Centers for Medicare and Medicaid Services campaign to decrease the use of antipsychotics in nursing homes nationwide, and the Nursing Home Compare website related to antipsychotics, it is clear that much work remains to be done. It is a promising sign however, that the government initiatives have influenced and increased research surrounding the topic. The majority of the studies included in this review were published between 2006 and 2012 after the black box warning was issued. Many of these studies focus on the facility characteristics included in the national OSCAR database, which surveys revenue sources, ownership, staffing, resident mix and deficiency reporting. The studies published after 2012, the year CMS launched an initiative to decrease use, investigate additional nursing home characteristics that influence APM use including: consultant psychiatry, pharmaceutical marketing, physical restraint deficiency reporting, and the presence of social services (Additional file 3). All of these studies provide valuable information about the use of APMs in the nursing home population in the U.S. Policy and management interventions to reduce the unnecessary use of APMs in elderly nursing home patients should focus on the facility and quality characteristics associated with use and address the root causes of excessive use of these dangerous drugs. The variation in use based upon staffing, market, physical, and quality characteristics of the nursing homes illustrates the need for a strict and systematic approach to APM use. Facility characteristics discussed in this review could inform meaningful and evidence-based interventions that would result in reduced use of APMs and the serious and poor health outcomes associated with their use.

#### International implications

The findings of this literature review can benefit an international audience, as high rates of APM use in nursing homes are common not only in the U.S. but in other nations as well. Although skilled nursing care can differ between nations, the facility characteristics affecting the use of APMs may be similar. Understanding the facility characteristics affecting use of these drugs in nursing homes can help inform policies and research internationally. It is crucial to consider the potential causes of overuse. In a campaign to reduce use in England, it was noted that although good practice guidelines are available, there has not been a clear integration into clinical practice [13]. Looking at the facility characteristics may help to provide insight into the difficulties of translating clinical guidelines into practice. It is important to look at international approaches to the use of APMs in the treatment of patients with dementia in order to find the safest and most effective treatment for this population.

### Limitations

This study has several important limitations. First, the studies included in the review did not sample the same population; some included national data and others data from a single state. Second, it was difficult to systematically judge the quality of the articles because observational studies of several varieties were included. Finally, the expert consultant panel used in the modified Delphi process was comprised of providers from limited geographical areas (Arizona, Washington, Pennsylvania, Illinois, South Carolina and Oregon). This may have resulted in APM use etiology suggestions consistent with nursing homes in those areas, which may have differed from other regions. Surveying health professionals across the nation would result in a more representative and potentially less biased perspective. Importantly, this review was constrained to facility level features, many of which are not remediable.

## Conclusion

The use of APMs in U.S. nursing homes was found to vary based on physical, staffing, occupancy, market, and quality characteristics. Due to the dangerous health outcomes associated with use of APMs in the elderly, it is critical that unnecessary use be reduced. The facility characteristics outlined in this review may serve as a basis for meaningful interventions to improve the quality of care in nursing homes across the nation and abroad.

## Additional file

Additional file 1: Database search strategies. (DOCX 32 kb)
Additional file 2: PRISMA checklist. (DOCX 22 kb)
Additional file 3: Table of included studies by time period. (DOCX 25 kb)

## Abbreviations

APM: Antipsychotic medication
BH: Behavioral health
CMS: Centers for medicare and medicaid services
FDA: Food and drug administration
LPN: Licensed practical nurse
MDS: Minimum data set
OSCAR: Online survey, certification and reporting
PRISMA: Preferred reporting items for systematic reviews and meta-analyses
RN: Registered nurse
STROBE: Strengthening the reporting of observational studies in epidemiology
U.S.: United States
