# Supplementary material for: Variation in use of antipsychotic medications in nursing homes in the United States: A systematic review
Source: BMC Geriatr. 2017 Jan 26;17:32. doi: 10.1186/s12877-017-0428-1 (PMC5267409; doi:10.1186/s12877-017-0428-1)
Supplement: Additional file 1: — Database search strategies. (DOCX 32 kb) [file 12877_2017_428_MOESM1_ESM.docx]

# **Additional file 1: Database search strategies**

## **Ovid/MEDLINE: Last Search conducted on 7/10/2015**

|  | **Searches** | **Results** |
| --- | --- | --- |
| 1 | exp Nursing Homes/ | 33196 |
| 2 | exp Homes for the Aged/ | 11580 |
| 3 | exp Long-Term Care/ | 22545 |
| 4 | 1 or 2 or 3 | 55866 |
| 5 | ((nursing or extended or long term or intermediate or skilled or elder* or senior* or geriatric or convalesce* or retirement or rest) adj2 (institution* or home* or facility* or unit* or center* or centre* or care)).mp. | 136286 |
| 6 | 4 or 5 | 141090 |
| 7 | (quetiapine or seroquel).mp. [mp=title, abstract, original title, name of substance word, subject heading word, keyword heading word, protocol supplementary concept word, rare disease supplementary concept word, unique identifier] | 3852 |
| 8 | (olanzapine or Zyprexa).mp. [mp=title, abstract, original title, name of substance word, subject heading word, keyword heading word, protocol supplementary concept word, rare disease supplementary concept word, unique identifier] | 7460 |
| 9 | (risperidone or risperdal).mp. [mp=title, abstract, original title, name of substance word, subject heading word, keyword heading word, protocol supplementary concept word, rare disease supplementary concept word, unique identifier] | 8183 |
| 10 | (clozapine or clozaril).mp. [mp=title, abstract, original title, name of substance word, subject heading word, keyword heading word, protocol supplementary concept word, rare disease supplementary concept word, unique identifier] | 10693 |
| 11 | (aripiprazole or Abilify).mp. [mp=title, abstract, original title, name of substance word, subject heading word, keyword heading word, protocol supplementary concept word, rare disease supplementary concept word, unique identifier] | 2755 |
| 12 | (ziprasidone or Geodon).mp. [mp=title, abstract, original title, name of substance word, subject heading word, keyword heading word, protocol supplementary concept word, rare disease supplementary concept word, unique identifier] | 1720 |
| 13 | exp Antipsychotic Agents/ | 106204 |
| 14 | antipsychotic*.mp. [mp=title, abstract, original title, name of substance word, subject heading word, keyword heading word, protocol supplementary concept word, rare disease supplementary concept word, unique identifier] | 54834 |
| 15 | 13 or 14 | 115350 |
| 16 | 7 or 8 or 9 or 10 or 11 or 12 or 15 | 118014 |
| 17 | 6 and 16 | 1490 |
| 18 | limit 17 to english | 1291 |
| 19 | from 18 keep 1-1291 | 1291 |

## **Elsevier/Embase: Last search conducted on 7/10/2015**

| No. | Query | Results |
| --- | --- | --- |
| #13 | **#11** AND (**'article'**/it OR **'review'**/it) AND [english]/lim | **2564** |
| #12 | **#11** AND (**'article'**/it OR **'review'**/it) | **2897** |
| #11 | **#9** AND **#10** | **3952** |
| #10 | **#1** OR **#2** OR **#3** OR **#4** OR **#5** | **175549** |
| #9 | **#6** OR **#7** OR **#8** | **254837** |
| #8 | **antipsychotic***:ab,ti OR (**anti** NEXT/1 **psychotic***):ab,ti | **43987** |
| #7 | **quetiapine**:ab,ti OR **seroquel**:ab,ti OR **olanzapine**:ab,ti OR **zyprexa**:ab,ti OR **risperidone**:ab,ti OR **risperdal**:ab,ti OR **clozapine**:ab,ti OR **clozaril**:ab,ti OR **aripiprazole**:ab,ti OR **abilify**:ab,ti OR **ziprasidone**:ab,ti OR **geodon**:ab,ti | **31325** |
| #6 | **'quetiapine'**/exp OR **'olanzapine'**/exp OR **'risperidone'**/exp OR **'clozapine'**/exp OR **'aripiprazole'**/exp OR **'ziprasidone'**/exp OR **'neuroleptic agent'**/exp | **248121** |
| #5 | ((**nursing** OR **extended** OR **'long term'** OR **intermediate** OR **skilled** OR **elder*** OR **senior*** OR **geriatric** OR **convalesce*** OR **retirement** OR **rest**) NEAR/2 (**institution*** OR **home*** OR **facility*** OR **unit*** OR **center*** OR **centre*** OR **care**)):ab,ti | **99896** |
| #4 | **'nursing home patient'**/exp | **3692** |
| #3 | **'long term care'**/mj | **16726** |
| #2 | **'elderly care'**/exp | **68253** |
| #1 | **'nursing home'**/exp | **44445** |

## **Wiley/Cochrane Library: Last search conducted on 7/07/2015**

| Search Name: antipsychotics in nursing homes |
| --- |
| Last Saved: 07/07/2015 22:42:18.696 |
| Description: |
|  |
| ID Search |
| #1 MeSH descriptor: [Nursing Homes] explode all trees |
| #2 MeSH descriptor: [Homes for the Aged] explode all trees |
| #3 MeSH descriptor: [Long-Term Care] explode all trees |
| #4 #1 or #2 or #3 |
| #5 ((nursing or extended or long term or intermediate or skilled or elder* or senior* or geriatric or convalesce* or retirement or rest) near/2 (institution* or home* or facility* or unit* or center* or centre* or care)):ti,ab,kw |
| #6 #4 or #5 |
| #7 (quetiapine or seroquel):ti,ab,kw (Word variations have been searched) |
| #8 (olanzapine or Zyprexa):ti,ab,kw (Word variations have been searched) |
| #9 (risperidone or risperdal):ti,ab,kw |
| #10 (clozapine or clozaril):ti,ab,kw |
| #11 (aripiprazole or Abilify):ti,ab,kw |
| #12 (ziprasidone or Geodon):ti,ab,kw |
| #13 MeSH descriptor: [Antipsychotic Agents] explode all trees |
| #14 antipsychotic*:ti,ab,kw |
| #15 #13 or #14 |
| #16 #7 or #8 or #9 or #10 or #11 or #12 or #15 |
| #17 #6 and #16 |

## **Thomson-Reuters/Web of Science: Last search conducted on 7/07/2015**

| # 7 | [872](http://apps.webofknowledge.com/summary.do?product=WOS&doc=1&qid=8&SID=2CxTcAFA5JrQlDzpDw7&search_mode=CombineSearches&update_back2search_link_param=yes) | #4 AND #1  **Refined by:** **DOCUMENT TYPES:** ( ARTICLE OR REVIEW OR CORRECTION ) AND **LANGUAGES:** ( ENGLISH )  Indexes=SCI-EXPANDED, SSCI, A&HCI, CPCI-S, CPCI-SSH, BKCI-S, BKCI-SSH, CCR-EXPANDED, IC Timespan=All years |
| --- | --- | --- |
| # 6 | [906](http://apps.webofknowledge.com/summary.do?product=WOS&doc=1&qid=7&SID=2CxTcAFA5JrQlDzpDw7&search_mode=CombineSearches&update_back2search_link_param=yes) | #4 AND #1  **Refined by:** **DOCUMENT TYPES:** ( ARTICLE OR REVIEW OR CORRECTION )  Indexes=SCI-EXPANDED, SSCI, A&HCI, CPCI-S, CPCI-SSH, BKCI-S, BKCI-SSH, CCR-EXPANDED, IC Timespan=All years |
| # 5 | [1,056](http://apps.webofknowledge.com/summary.do?product=WOS&doc=1&qid=6&SID=2CxTcAFA5JrQlDzpDw7&search_mode=CombineSearches&update_back2search_link_param=yes) | #4 AND #1  Indexes=SCI-EXPANDED, SSCI, A&HCI, CPCI-S, CPCI-SSH, BKCI-S, BKCI-SSH, CCR-EXPANDED, IC Timespan=All years |
| # 4 | [54,491](http://apps.webofknowledge.com/summary.do?product=WOS&doc=1&qid=5&SID=2CxTcAFA5JrQlDzpDw7&search_mode=CombineSearches&update_back2search_link_param=yes) | #3 OR #2  Indexes=SCI-EXPANDED, SSCI, A&HCI, CPCI-S, CPCI-SSH, BKCI-S, BKCI-SSH, CCR-EXPANDED, IC Timespan=All years |
| # 3 | [36,912](http://apps.webofknowledge.com/summary.do?product=WOS&doc=1&qid=4&SID=2CxTcAFA5JrQlDzpDw7&search_mode=AdvancedSearch&update_back2search_link_param=yes) | TS=(antipsychotic*)  Indexes=SCI-EXPANDED, SSCI, A&HCI, CPCI-S, CPCI-SSH, BKCI-S, BKCI-SSH, CCR-EXPANDED, IC Timespan=All years |
| # 2 | [32,765](http://apps.webofknowledge.com/summary.do?product=WOS&doc=1&qid=2&SID=2CxTcAFA5JrQlDzpDw7&search_mode=AdvancedSearch&update_back2search_link_param=yes) | TS=(quetiapine OR seroquel OR olanzapine OR Zyprexa OR risperidone OR Risperdal OR clozapine OR clozaril OR aripiprazole OR Abilify OR ziprasidone OR Geodon)  Indexes=SCI-EXPANDED, SSCI, A&HCI, CPCI-S, CPCI-SSH, BKCI-S, BKCI-SSH, CCR-EXPANDED, IC Timespan=All years |
| # 1 | [95,408](http://apps.webofknowledge.com/summary.do?product=WOS&doc=1&qid=1&SID=2CxTcAFA5JrQlDzpDw7&search_mode=AdvancedSearch&update_back2search_link_param=yes) | TS=((nursing or extended or long-term or intermediate or skilled or elder* or senior* or geriatric or convalesce* or retirement or rest) NEAR/2 (institution* or home* or facility* or unit* or center* or centre* or care))  Indexes=SCI-EXPANDED, SSCI, A&HCI, CPCI-S, CPCI-SSH, BKCI-S, BKCI-SSH, CCR-EXPANDED, IC Timespan=All years |

## **EBSCO/PsycINFO: Last search conducted on 7/10/2015**

The search below was run on 7/7/2015. On 7/10/16 it was rerun but limited to Articles in English and limited to Academic Journals and Dissertations with a total of 2,541 citations retrieved.

| **#** | **Query** | **Results** |
| --- | --- | --- |
| S9 | S7 AND S8 | 2,748 |
| S8 | S4 OR S5 OR S6 | 34,877 |
| S7 | S1 OR S2 OR S3 | 183,477 |
| S6 | antipsychotic* | 22,928 |
| S5 | DE "Neuroleptic Drugs" | 17,809 |
| S4 | (quetiapine OR seroquel OR olanzapine OR Zyprexa OR risperidone OR Risperdal OR clozapine OR clozaril OR aripiprazole OR Abilify OR ziprasidone OR Geodon) | 16,315 |
| S3 | ((nursing OR extended OR long-term OR "long term" OR intermediate OR skilled OR elder* OR senior* OR geriatric OR convalesce* OR retirement OR rest) N2 (institution* OR home* OR facility* OR unit* OR center* OR centre* OR care)) | 183,477 |
| S2 | DE "Long Term Care" | 3,702 |
| S1 | DE "Nursing Homes" | 7,066 |

## **EBSCO/CINAHL: Last search conducted on 7/10/2015**

The search below was run on 7/7/2015. On 7/10/16 it was rerun but limited to Articles in English and limited to Academic Journals and Dissertations with a total of 636 citations retrieved.

| **#** | **Query** | **Results** |
| --- | --- | --- |
| S11 | S5 AND S9 | 778 |
| S10 | S5 AND S9 | 784 |
| S9 | S6 OR S7 OR S8 | 13,721 |
| S8 | antipsychotic* | 11,223 |
| S7 | (MH "Antipsychotic Agents") OR (MH "Olanzapine") OR (MH "Risperidone") OR (MH "Quetiapine") OR (MH "Aripiprazole") OR (MH "Clozapine") | 11,647 |
| S6 | (quetiapine OR seroquel OR olanzapine OR Zyprexa OR risperidone OR Risperdal OR clozapine OR clozaril OR aripiprazole OR Abilify OR ziprasidone OR Geodon) | 5,319 |
| S5 | S3 OR S4 | 156,417 |
| S4 | ((nursing OR extended OR long-term OR intermediate OR skilled OR elder* OR senior* OR geriatric OR convalesce* OR retirement OR rest) N2 (institution* OR home* OR facility* OR unit* OR center* OR centre* OR care)) | 155,365 |
| S3 | S1 OR S2 | 40,883 |
| S2 | (MH "Long Term Care") | 20,050 |
| S1 | (MH "Nursing Homes+") OR (MH "Nursing Home Patients") | 27,095 |

## **ProQuest/Sociological Abstracts: Last search conducted on 7/7/2015**

((SU.EXACT("Nursing Homes") OR SU.EXACT("Long Term Care")) OR ((nursing OR extended OR long-term OR “long term” OR longterm OR intermediate OR skilled OR elder* OR senior* OR geriatric OR convalesce* OR retirement OR rest) NEAR/2 (institution* OR home* OR facility* OR unit* OR center* OR centre* OR care))) AND ((quetiapine OR seroquel OR olanzapine OR Zyprexa OR risperidone OR Risperdal OR clozapine OR clozaril OR aripiprazole OR Abilify OR ziprasidone OR Geodon) OR (antipsychotic* OR anti-psychotic*))
