# Supplementary material for: Variation in use of antipsychotic medications in nursing homes in the United States: A systematic review
Source: BMC Geriatr. 2017 Jan 26;17:32. doi: 10.1186/s12877-017-0428-1 (PMC5267409; doi:10.1186/s12877-017-0428-1)
Supplement: Additional file 3: — Table of included studies by time period. (DOCX 25 kb) [file 12877_2017_428_MOESM3_ESM.docx]

| **Additional file 3: Table of included studies by time period** | | |
| --- | --- | --- |
| **Studies 2005 or earlier** | | |
| **Author** | **Study Title** | **Facility Characteristic Associated with APM Use** |
| Briesacher BA, Limcangco MR, Simoni-Wastila L, et al., 2005 | The quality of antipsychotic drug prescribing in nursing homes | NH characteristics were comparable for both APM user and non-users (ownership, no. of beds) except for residence in the South where antipsychotic rates were lower (31.1% vs 38.3; P<.001) |
| Svarstad BL, Mount JK, Bigelow W, 2001 | Variations in the treatment culture of nursing homes and responses to regulations to reduce drug use | High nurse-to-resident staffing ratio associated with greater reduction of APM use |
| **2006-2012** | | |
| Bowblis JR, Crystal S, Intrator O, et al., 2012 | Response to regulatory stringency: the case of antipsychotic medication use in nursing homes | Facilities in states that issue more deficiency citations have higher use of antipsychotics |
| Castle NG, Hanlon JT, Handler SM, 2009 | Results of a longitudinal analysis of national data to examine relationships between organizational and market characteristics and changes in antipsychotic prescribing in US nursing homes from 1996 through 2006 | For-profit facilities were more likely to increase APM use (AOR, 1.58; 95% CI, 1.51–1.65; P ≤ 0.001). Members of chains were less likely to increase APM use (AOR, 0.82; 95% CI, 0.79–0.85; P ≤ 0.001). Higher levels of competition (AOR, 1.22; 95% CI, 1.16–1.29; P ≤ 0.001), and a higher Medicaid reimbursement rate (AOR, 0.88; 95% CI, 0.85–0.92; P ≤ 0.001) were associated with decreasing use of APMs. |
| Chen Y, Briesacher BA, Field TS, et al., 2010 | Unexplained variation across US nursing homes in antipsychotic prescribing rates | A higher proportion of high prescribing NHs  were more likely to be located in the South and have fewer  than 250 residents. A higher proportion of Q1 NHs were  located in the Midwest and had more than 250 residents. |
| Hughes CM, Lapane KL, Mor V, 2000 | Influence of facility characteristics on use of antipsychotic medications in nursing homes | The presence of special care units and mental health professionals were associated with increased antipsychotic use (beta = 1.70, SE = 0.23; beta = 0.24, SE = 0.17, respectively). Being located was associated with increasing use. Increasing size, being part of a chain, lower Medicaid census, higher occupancy rates, higher nurse aide staffing and higher RN staffing were associated with decreased APM use. |
| Huybrechts KF, Rothman KJ, Brookhart MA, et al., 2012 | Variation in antipsychotic treatment choice across US nursing homes | Located in urban area, larger facilities, facilities with Alzheimer special care units, with team-based physician care and mental health staff were more likely to prescribe atypical APMs. Facilities in south tended to prescribe conventional and in the northeast atypical. |
| Kamble P, Chen H, Sherer J, Aparasu RR, 2008 | Antipsychotic drug use among elderly nursing home residents in the United States | Enabling factors such as payment source and facility characteristics were not significantly associated with antipsychotic drug use |
| Kamble P, Chen H, Sherer J, Aparasu R, 2009 | Use of antipsychotics among elderly nursing home residents with dementia in the US: an analysis of National Survey Data | Bed capacity and APM use: 3-49 (OR=1), 50-99 (OR=0.78), 100-199 (OR=0.89), ≥200 (OR=0.88) |
| Kamble P, Sherer J, Chen H, Aparasu R, 2010 | Off-label use of second-generation antipsychotic agents among elderly nursing home resident. | Positively associated with off label use: nonprofit status (AOR 1.5) self-pay (AOR 1.7) for nursing home care Negatively Associated: Medicaid benefits (AOR 0.6) |
| Lester P, Kohen I, Stefanacci RG, Feuerman M, 2011 | Antipsychotic drug use since the FDA black box warning: survey of nursing home policies | For-profit facilities had higher rates of antipsychotic use (P=.0064). Facilities located in the western US were more likely to have a policy about APM use. |
| Miller SC, Papandonatos G, Fennell M, Mor V, 2006 | Facility and county effects on racial differences in nursing home quality indicators | For-profit facilities had higher odds of antipsychotic drug use irrespective of the proportion of AAs. The use of antipsychotic drug use increased as racial diversity increased and as nurse staffing decreased. Nursing staff ratios |
| Stevenson DG, Decker SL, Dwyer LL, et al., 2010 | Antipsychotic and benzodiazepine use among nursing home residents: findings from the 2004 National Nursing Home Survey | Facility traits predictive of greater odds of antipsychotic use among individuals who had no appropriate indication included greater facility share of Medicaid residents, lower facility share of Medicare residents, and being located in the Northeast (relative to the West of Midwest) and in a metropolitan area |
| Studies after 2012 | | |
| Bonner AF, Field TS, Lemay CA, et al., 2015 | Rationales that providers and family members cited for the use of antipsychotic medications in nursing home residents with dementia | Staff and leaders of NH identified social services with lower APM use. Staff and leaders of NH with high APM use identified consultant psychiatry more often than staff from lower-use facilities as having an influence. NHs with high antipsychotic use was more frequently located in census tracts of increased racial diversity. |
| Briesacher BA, Tjia J, Field T, et al., 2013 | Antipsychotic use among nursing home residents | Prevalence of APM prescribing in NHs varied significantly (quintile 1 vs quintiles 2-5, P<.001) with the highest quintile states located in the central south (28.1%; 95% CI, 27.0%-29.1%) and the lowest quintile states located mainly in the west 1(7.2%; 95% CI, 16.3%-18.1%). |
| Konetzka, R. Tamara, et al., 2014 | The effects of public reporting on physical restraints and antipsychotic use in nursing home residents with severe cognitive impairment | Antipsychotic use in the same residents increased more in NHs that were subject to public reporting of physical restraint use (4.5 vs 2.9 percentage points, P < .001). Approximately 36% of the increase in antipsychotic use may be attributable to public reporting of physical restraints. |
| Lucas JA, Chakravarty S, Bowblis JR, et al., 2014 | Antipsychotic medication use in nursing homes: a proposed measure of quality | Non-profit (OR=0.93 p <.01) and government facilities (OR=0.81 p <.01) were less likely to provide APM for residents without approved indications. Facilities with the highest proportion of Medicaid-funded residents, relative to the lowest, were 25-32% more likely to provide APMs. Higher levels of RN Staffing were associated with lower odds of APM use. Mental health staffing and medical teams showed no effects. Facilities within highest quartile of deficiencies had higher odds of APM use (OR=1.09). Facilities within highest quartiles of restraint use had higher odds of APM use. |
| Pimentel CB, Donovan JL, Field TS, et al., 2015 | Use of atypical antipsychotics in nursing homes and pharmaceutical marketing | There was minimal difference in APM use between facilities reporting having an encounter with pharmaceutical marketing and no contact. |
| Tjia J, Field T, Lemay C, et al., 2014 | Antipsychotic use in nursing homes varies by psychiatric consultant | Most of the NHs served by the 2 lowest ranked psychiatric consultant groups in our study had observed antipsychotic use below predicted levels, whereas half of the NHs served by the highest-ranked psychiatric consultant group had observed antipsychotic use above levels predicted for on-label indications |
